# Quinine

**Published:** 1871-06

**Authors:** Charles S. Sheldon

**Affiliations:** Winona


### Quinine.

BY CHARLES S. SHELDON, M. D., WINONA

Read before the Winona County Medical Society, May 1, 1871.

The subject I have chosen is one familiar to all. We have used this remedy daily for years, and have given more or less of study and thought to its proper administration and action in disease.

In treating this subject I do not expect to give you any discoveries, or even theories, of my own, but shall endeavor simply to review the ground gone over by others.

I shall consider briefly its action and therapeutic use.

We cannot devote too much attention to the subject of therapeutics. Diagnosis and pathology have of late made great advances, and perhaps before long we shall know what we *have* to cure; but we have to admit that in the understanding of the action and agency of medicines in the cure of disease, we are not so very much superior to our ancestors. We should rise to a higher degree of enlightenment, when we discard the empirical, and adopt the rational system of therapeutics. This can only be, till on the one hand, by an accurate knowledge of the symptoms of disease, we can meet each by its appropriate remedy, and, on the other hand, by a more definite acquaintance with the general action of a medicine, we may use it with greater skill and effect, and apply it even in cases where it has not yet proved beneficial. Especially should we study a remedy so generally used and so highly esteemed as quinine. Not a day passes but that we meet cases or conditions where its use is indicated. Questions relating to its absolute or relative efficiency in in these cases, its mode of action in curing disease, its dose and manner of administration, daily suggest themselves to us, and claim our careful attention. Here in Minnesota, these considerations are especially important. We have among us those who have formerly lived under malarious influences, and there is, undoubtedly, more or less of malaria in the State.. It therefore requires a nicer discrimination to ascertain those cases when quinine is indicated, and in what way to give it, than in Illinois, used heroically for ague, or in New England, used merely for its tonic effects.

As to the mode of action of quinine, there has always been, and there is now, a great diversity of opinion. Its tonic effects in some cases, its stimulant effect in others, and its sedative effects in still others, have led authors to take very different and opposite grounds. The views here presented are condensed from Headland, in his work on the action of medicines, often in his own words. I am aware that they will be met with disfavor by many, especially by those who have adopted the theory which regards the nerves as the prime factor in the production of most diseases; but they are, at least, worthy of consideration. He considers quinine as an hæmatic, and as being restorative rather than catalytic, i. e., a medicine which acts primarily in the blood, to restore some material wanting, rather than to destroy a poison existing. He asks, and attempts to answer, the following questions:

1. "*Does quinine act primarily in the blood, or on the nerves, and is its action of a permanent character?*"

2. "*Is it naturally present in the blood, or is there in the blood a substance which resembles it?*"

3. "*Does it remain in the blood, or is it wholly excreted?*"

4. "*If acting in the blood, does it effect a cure by supplying to it a material wanting, or by counteracting in it a morbid process?*"

There is no doubt but that it enters the blood, since it can be detected in that fluid after its administration. Once there, does it produce its tonic and anti-periodic effects by improving the blood, and so the whole system, or does it at once, and in the first place, act on the nerves? Is it an hæmatic or a neurotic? He believes it is *primarily* a neurotic, for the following reasons: Other nerve medicines are distinguished by an action which rapidly follows their administration. This action is not permanent, but rapidly passes away. It takes place in health as well as disease; as in the use of alcohol and digitalis. Most neurotics are capable of producing action by mere external contact with the nerves. They are chiefly used when the nervous system is unusually excited or depressed, and are of no permanent benefit in diseases depending upon blood disorded.

The action of hæmatics, on the contrary, is of an opposite kind in all these particulars. When we consider the primary action of quinine, too, we must conclude that it more resembles hæmatics, and that its influence on the nervous and muscular systems is secondary. Judging from analogy in the use of neurotics and astringents, we could not expect that any permanent improvement could be effected in either nerves or muscles, without its first acting in the blood. To make the case clearer, it can be shown that the diseases in which quinine is most used, are blood diseases. *Debility* seems primarily to be due to a want in the blood, which impairs the nutrition of the nerves and other organs, and thus interferes with the performanee of their functions. It follows fevers and accompanies chronic diseases, in both of which cases the blood has been exhausted by continual waste and excretion. I know that some will take exception to this doctrine. Intermittent fever, too, is now considered by most a blood disease. We might infer this from the analogy of other fevers; but we know, in addition, that it is caused by a peculiar poison, which produces in the blood a process like fermentation, causing periodical paroxysms. In the progress of the disease, too, the blood rather than the nerver, seems to be concerned. We have first a chill, then a hot stage, and then sweating. It would seem, then, as if the poison were eliminated in the sweating; but after working in the blood for a definite period, the same symptoms recur. The results of the disease, also, are evidence of its true nature. Long continued ague causes general anæmia, and an enlargement of the spleen, which could only be produced by a faulty condition of the circulation. In typhoid and typhus fevers, in scrofulous and tuberculous diseases, and in fact in all affections where detoriation of the blood is a known cause of disease, quinine, in tonic doses, has the most beneficial effect. The periodical attacks of neuralgia, benefitted by the use of quinine, are generally malarial in their origin, and so due primarily to a poisoned condition of the blood. Another fact pointing in the same direction, is, that ague is often, if not always connected with derangement of the liver. I think these positions are, in the main, well taken by the author, and the probabilities very strong that quinine is *primarily* an hæmatic.

We now come to the next question: *Is it naturally in the blood, or is there in healthy blood any substance which resembles it?* that is, is it a restorative hæmatic? for to act as a restorative, it must take the place of something which should be present in healthy blood. Although the bitter principle of the bile seems to possess a similar character to quinine, it is only till recently that we have found proof of the existence in the blood, of a substance similar to quinine. Dr. Dupre and Dr. Bence Jones having discovered quinine in the blood of a Guinea-pig to which it *had* been administered, found precisely the same reactions in a pig to which it had *not* been given. These experimonts were carefully conducted, and were conclusive as to the presence in the blood and tissues of a substance chemically identical with quinine. This they have named *animal quinoidine.* If this be not quinine itself, we may at least conclude that the presence of quinine in the blood, would not be unnatural to it.

THIRDLY.—*Is quinine wholly excreted from the blood?* If not, we may conclude that it remains in it, and so can act as a restorative. From many experiments it has been proved conclusively that it does remain in the blood, i. e., when given in a small dose, it is not excreted at all, but when given in an excessive dose it makes its appearance in the urine, like other restorative medicines.

FINALLY.—*Does it improve the condition of the blood when deficient in any of its natural materials,* i. e., *does it act as a restorative in supplying something wanting, or as a catalytic in counteracting something present?* The fact that it is not unnatural to the blood, and is not always excreted from it, is in favor, *a priori,* of its being a restorative. Besides, a catalytic has some peculiar action on the blood in health, but a restorative, in moderate doses, none. On all these grounds we must consider quinine a restorative. Compare quinine with arsenic, a catalytic, and we find they differ in all these particulars. and also in their use; quinine being used in debility and when there is some want in the system, arsenic in lepra and kindred skin diseases, caused by some morbid agency. To be sure both are used in ague, but perhaps this disease is curable either by supplying something, or by neutralizing something else.

If these points have been well made, we must conclude that quinine is a restorative hæmatic, and a presumption is established that in those diseases where it is curative, there is some deficiency in the blood, which can be supplied by it. It may be that this is simply a *theory,* but we accept many medical ideas as facts, which have really fewer grounds for our belief. These views are presented on the supposition that they might be new to some, and with the hope that their presentation might excite a more earnest spirit of inquiry and discussion regarding such an important topic.

Therapeutically considered, quinine is certainly one of our most valuable remedies, not only from its positive, but also from its negative virtues; for while other medicines of equal power and value, are often harmful and destructive in their character, I think we may justly class quinine among the most harmless and safe of our remedies. It is a *remedy,* and not a *poison;* and while opium and mercury have killed many, and destroyed the constitutions of more,

I can find no case where quinine has produced lasting harmful results. Not but that it may be injudiciously used, especially in acute diseases, but that, on the whole, there is less danger in its use than in any other remedy of equal power. It is very unfortunate that there exists such a strong prejudice against it in the popular mind, many persons being unwilling to take it, and its beneficial effects being lessened in the case of others by their suspicion of its injurious nature. It is difficult to account for such popular delusion; but we know that many confound cause and effect in cases of long continued ague, accompanied with the use of quinine, and that a large proportion of the community, including well educated professional men, are so grossly ignorant of its character as to suppose it a preparation of mercury or opium. We can only wait and hope that this and kindred delusions will be dissipated by time, and a greater enlightenment of those who have a *moderate* stock of common sense on topics *disconnected* with medicine. Although there is no disease in which quinine acts so favorably and surely as in malarial fevers, there is no doubt of its value in a vast number of affections. In intermittent fever the profession now favors large doses in the interval of the paroxysms, the rule being to give enough to produce a moderate degree of quinism. To stop a single paroxysm, Prof. Alonzo Clark gives gr. j. of opium and gr. x. of capsicum, with the quinine. In intermittent neuralgia all authors agree that large doses, gr. xx. to gr. xxx. daily, for a week or more, will afford the best results in most cases.

The use of quinine in typhoid fever has given rise to much discussion, some claiming for it the virtues of a specific, and others stigmatising it as highly injurious. Stille concludes that it is a remedy of secondary value here, even if it possesses any virtues at all. Wood has never found in it any other favorable influence than a moderate supporting effect in the low states of the disease. Prof. Clark does not mention its use in this affection. Aitken simply says it is recommended by Dr. Murchison in half grain doses. Flint advises its use, as a tonic, in small doses. Headland thinks it admissible in all fevers, care being taken to give when the pulse is soft and the skin and tongue moist. Liebermeister, basing his belief on a long and carefully conducted series of experiments, thinks it has actual curative properties, in reducing the fever, and ameliorating the other symptoms. I think we may safely conclude, from our daily experience, that it cannot claim a directly curative influence, it certainly has a tonic and supporting power, and that it will be safe and useful to give, in small doses, in nearly every stage of the disease.

In several diseases of the digestive organs it is of much service. In atonic dyspepsia, we often get excellent results from its use, either alone or with other remedies. In the night-sweats of phthisis, with sulphuric acid, it is especially serviceable, and all agree that a profuse sweating during sleep, in *any* disease, is an indication for its use. In convalescence from most fevers, and in all cases of general debiliiy, it is indicated.—*Northwestern Med. and Surgical Journal.*

